# Improvement in Electrode Performance of Novel SWCNT Loaded Three-Dimensional Porous RVC Composite Electrodes by Electrochemical Deposition Method

**DOI:** 10.3390/nano8010019

**Published:** 2018-01-01

**Authors:** Ali Aldalbahi, Mostafizur Rahaman, Mohammed Almoigli, Al Yahya Meriey, Khalid N. Alharbi

**Affiliations:** 1 Department of Chemistry, College of Science, King Saud University, Riyadh 11451, Saudi Arabia; mrahaman@ksu.edu.sa (M.R.); alyahya@gmail.com (A.Y.M.); 2 Nuclear Sciences Research Institute, King Abdulaziz City for Science and Technology, Riyadh 11442, Saudi Arabia; almoiqli@kacst.edu.sa (M.A.); kalharbi@kacst.edu.sa (K.N.A.)

**Keywords:** SWCNT/RVC composites, morphology, cyclic voltammetry, electrochemical deposition, electrode stability

## Abstract

The three-dimensional (3D) composite electrodes were prepared by depositing different amounts of acid-functionalized single-walled carbon nanotubes (a-SWCNTs) on porous reticulated vitreous carbon (RVC) through the electrochemical deposition method. The SWCNT was functionalized by the reflux method in nitric acid and was proven by Raman and visible spectra. The optimum time for sonication to disperse the functionalized SWCNT (a-SWCNT) in dimethyl formamide (DMF) well was determined by UV spectra. The average pore size of RVC electrodes was calculated from scanning electron microscopy (SEM) images. Moreover, the surface morphology of composite electrodes was also examined by SEM study. All 3D electrodes were evaluated for their electrochemical properties by cyclic voltammetry. The result showed that the value of specific capacitance of the electrode increases with the increase in the amount of a-SWCNT in geometric volume. However, the value of specific capacitance per gram decreases with the increase in scan rate as well as the amount of a-SWCNT. The stability of the electrodes was also tested. This revealed that all the electrodes were stable; however, lower a-SWCNT-loaded electrodes had excellent cyclic stability. These results suggest that the a-SWCNT-coated RVC electrodes have promise as an effective technology for desalination.

## 1. Introduction

The major requirements for the materials to be used as electrodes are good polarizability, low electrical resistance, high surface area and capacitance, no participation in faradaic reactions at the applied voltage, high porosity and conductivity [1]. This means that the correct choice of material is the most important issue for making high performance electrodes [2]. There are a number of different porous carbon materials that can be applied as electrodes such as carbon felt [3], carbon aerogels [4], carbon cloth [5] and titania-modified carbon cloth [6]. More recently, carbon nanotube structures [7], carbon nanofiber [8] and inherently conducting polymers such as polypyrrole [9] have been investigated for this purpose.

Carbon nanotubes (CNTs) have many impressive characteristics such as high electrical conductivity, mechanical strength, optical transparency and thermal stability [10,11]. These properties have attracted great attention, produced significant activities and opened a new window in many areas of science and engineering [10,12]. In particular, carbon nanotubes (CNTs) have been used as electrode materials because they afford novel additional functional properties, such as structure, surface area, activity and conductivity [13,14]. These electrodes were made to be used as two-dimensional (2D) sheet electrodes. However, to get more effective electrodes, one of the effective ways is that CNTs electrodes could be constructed as three-dimensional (3D) structures. The advantages of this approach are high porosity, increased surface area and efficient pathways for ion diffusion [15].

Reticulated vitreous carbon (RVC) has a three-dimensional (3D) structure, and it can be used as a platform to support carbon materials. The RVC has been used in many applications such as in bony prostheses [16], heart valves [17], molecular sieves [18], absorption electrodes [19], cathode materials in order to recover Co^2+^ ions [20] and cation exchangers for the adsorption of Ca^2+^ ions from aqueous solutions [21]. It has also been applied in many primary and secondary battery systems [22,23], as a porous cathode for the production of hydrogen gas from flowing alkaline solution [24] and a viable field emission electron source [25]. Furthermore, the low density and high electrical conductivity of RVC are very attractive for electrochemical applications as three-dimensional electrodes [26]. The structure of RVC lends itself to easy surface modification with various materials [27,28].

In this article, commercial SWCNT were functionalized to introduce carboxylic acid groups by acid treatment in order to facilitate dispersion in DMF and to convert SWCNT into hydrophilic materials to reduce the flow resistance of aqueous solutions through the electrodes. The SWCNT were characterized using Raman and visible spectra. This acid-treated SWCNT (a-SWCNT) were dispersed in DMF by sonication, and the sonication time was optimized. In addition, the energy of sonication of each mg of a-SWCNT material was determined. The purpose of forming a-SWCNT solutions was in order to coat and fill the RVC electrode, which was cleaned by dilute acid. Here, the RVC electrode is used to support a-SWCNT coating for use as a 3D electrode. a-SWCNT material-coated RVC electrodes, with various coating levels up to filled electrode pores, were characterized for their morphology and electrochemical properties. Moreover, the cyclic stability of the electrodes was also tested.

## 2. Materials, Methods and Experimental Section

### 2.1. Chemicals and Materials

Commercial single-walled carbon nanotubes (Hipco-CCNI/Lot # p1001) were purchased from Carbon Nanotechnologies, Inc. (Houston, TX, USA) and were used as received. The following chemicals obtained from Sigma-Aldrich (Darmstadt, Germany) were also used as received: *N*,*N*-dimethylformamide (DMF) (analytical reagent (AR) grade), concentrated nitric acid (70%) and sodium chloride (AR grade). The reticulated vitreous carbon (compressed at 60, 45 and 30 ppi (pores per inch)) was purchased from ERG Materials and Aerospace Engineering (Oakland, CA, USA) and cleaned before use. Membrane filters (0.2 µm GTTP) were purchased from Millipore (Sigma-Aldrich, Darmstadt, Germany) and were used as received. Milli-Q water with a resistivity of 18.2 mΩ·cm^−1^ was used in all preparations.

### 2.2. Functionalization of Carbon Nanotubes

As-received SWCNT material was functionalized by a reflux method. In a typical procedure, 20 mg of the material were transferred into a round-bottom flask filled with 40 mL of 6 M nitric acid (HNO_3_) and equipped with a magnetic stirring bar and a reflux condenser. The flask was immersed in an oil bath at a temperature of 120 °C for 6 h. The acid treated SWCNT was then filtered and washed with water until the filtrate was neutral, then washed with 10 mL of methanol, followed by 10 mL of DMF. The functionalized material, henceforth referred to as acid-treated SWCNT (a-SWCNT), was dried in an oven at a temperature of 105 °C for 48 h.

### 2.3. Dispersion of a-SWCNT

A dispersion of 15 mL containing a-SWCNT 0.2% *w*/*v* in *N*,*N*-dimethylformamide (DMF), one of the best solvents reported for SWCNT dispersions [29], was obtained by using a Branson homogenizer (Branson Ultrasonics Corporation, Danbury, CT, USA), Sonifier Model S-450D, equipped with a 13-mm step disruptor horn and a 3-mm tapered microtip, operating at 20 kHz frequency and 25% amplitude as the power output (100 W), with a pulse of two seconds on and one second off. The dispersion was sonicated in a water-ice bath to prevent the suspension from overheating. Homogeneity and birefringence of the dispersion were checked using an Olympus BH-2 microscope (Olympus Optical Co. Ltd., Tokyo, Japan) in transmission mode between crossed polarizers. In addition, the optimization of sonication time, sonication energy and solution stability will be discussed in the results and discussion section.

### 2.4. Pre-Treatment of the RVC Electrode

The RVC electrodes with a length of 4 cm, a width of 1.8 cm and a thickness of 0.3 cm (2.16 cm^3^) were cut from a block of RVC material, and the impurities were removed by soaking in 2M HNO_3_ for 24 h [30]. The acid from the electrodes was then removed by washing several times with distilled water. The removal of organic impurities from the RVC electrodes was done by soaking in methanol for 2 h [30]. The electrodes were then weighed after drying with the help of nitrogen flow and heated in an oven overnight at 110 °C.

### 2.5. a-SWCNT Dip Coated RVC Electrode

**Scheme 1:**
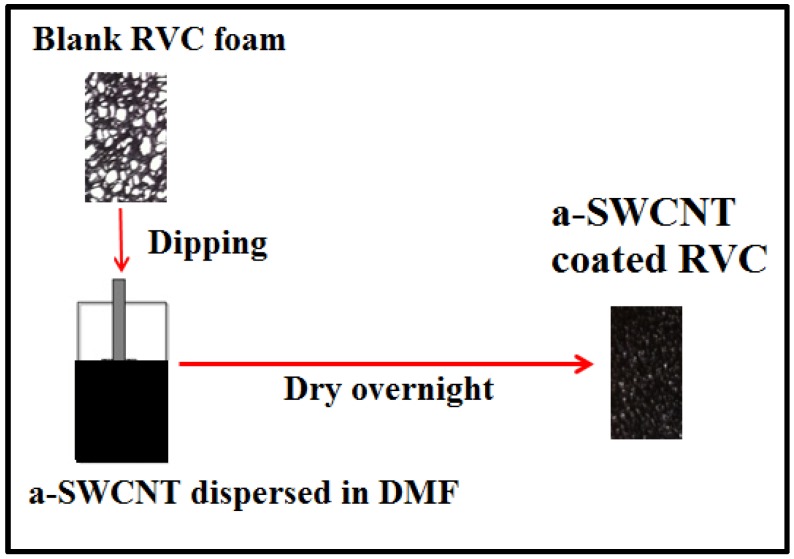
The full process of dip coating reticulated vitreous carbon (RVC) in acid-functionalized single-walled carbon nanotube (a-SWCNT) solution.

The dip-coating method, which was used in our work, was kept simple and low cost [31]. All RVC electrodes, which have 2.16 cm^3^ volumes, were immersed slowly into the 0.2% *w*/*v* a-SWCNT solution to allow the air to escape and prevent the formation of air pockets; during this time, the solution was seen to fill the whole RVC electrode via capillary action within a few seconds. After that, all electrodes were removed from the solution to dry them at room temperature before drying them in an oven at 100 °C for 2 h. This procedure was repeated several times to study the maximum loading level of a-SWCNT on the RVC substrate. Scheme 1 shows a schematic diagram of the full process of dip-coating RVC in a-SWCNT solution. The process of the RVC electrode being soaked in solution and drying afterward in an oven overnight is shown schematically. The substrate, after dip coating, was dried at 100 °C in an oven for 2 h and then in a vacuum oven at 50 °C for 2 h to remove all organic solvents remaining in the micropores of the electrode. The weights of a-SWCNT loadings on the RVC electrodes were determined by weighing the electrodes before and after dip coating.

### 2.6. Electrochemical Characterization

The capacitance of a-SWCNT was determined by cyclic voltammetry (CV). An a-SWCNT/RVC composite electrode was used as the working electrode (WE) in 1 M NaCl aqueous solution and scanned in the voltage range between −0.2 and 1.0 V using a three-electrode system; the RVC electrode and Ag/AgCl (3 M NaCl) were used as the counter electrode (CE) and the reference electrode (RE), respectively. The scan rates used were 5, 10, 20, 50, 100 and 200 mV/s, respectively. Contacts to the WE and CE were made using Pt wire. The electrochemical impedance spectra (EIS) for RVC and RVC/a-SWCNT composite electrode were measured at the angular frequency range 10^1^–10^8^ Hz with the potential amplitude of 10 mV.

### 2.7 Physical Characterization

The morphology of the a-SWCNT-coated RVC electrodes was analyzed by the use of a field emission scanning electron microscope (FESEM, ZEISS Sigma, Hamburg, Germany) at a specific voltage 0.5 KV. Furthermore, a-SWCNT functionalization was characterized by: (1) visible absorption spectra obtained using quartz cuvettes and (2) Raman spectra obtained with a Raman spectrometer (Jobin Yvon Horiba HR800 Raman spectrometer, Kyoto, Japan) equipped with a visible Raman microscope and charged-couple device (CCD) detector. The excitation wavelength was 632.81 nm, and spectra were obtained over 30 s at 1.0 cm^−1^ resolution.

## 3. Results and Discussion

### 3.1. Characterization of SWCNT after Functionalization by Raman Spectroscopy

Raman spectroscopy was used to investigate the SWCNT surface after acid treatment. Raman spectroscopy is a powerful technique for the characterization of the structure of carbon nanotubes [32]. It is known that the general characteristics of a Raman spectrum of CNTs have two bands appearing at 1582 cm^−1^ (G band) and 1350 cm^−1^ (D band). The shape and intensity of the D band corresponds to the sp^3^ hybridized carbon atoms, and the G band is associated with the tangential vibration modes of CNT sidewall C–C bonds. The intensity ratio (*R*) of these D and G peaks (I_D_/I_G_) is often used to estimate the quality of the structure of the CNT sample [33,34]. In addition, the dominant second-order feature in SWCNT Raman spectra is the G’ band, which is located at about 2600 cm^−1^, and this is sensitive to charge transfer effects due to structural modifications of the nanotube walls induced by the attachment of different chemical species. Furthermore, the M band appearing at about 1755 cm^−1^ is a second-order peak tentatively assigned to a combination mode of the G and the radial breathing mode (RBM) bands [32,33]. The radial breathing mode (RBM) can be used to determine the nanotube diameter (*d_t_*) through its frequency (*ω*RBM) [35,36]. These features are unique to carbon nanotubes and occur at frequencies between 120 and 350 cm^−1^ for SWCNT for diameters in the range *ω*RBM *=* A*/d_t_ +* B, where A = 234 cm^−1^ nm and B = 10 cm^−1^, and where B is an upshift in *ω*RBM assigned to tube-tube interactions. For typical SWNT bundles in the diameter range *d_t_* = 1.5 ± 0.2 nm and if the nanotube diameter is greater than 2 nm, the RBM spectrum is difficult to observe [32,35,36]. Hence, the mean diameter of SWCNT can be calculated by Equation (1);

*d_t_* = 248/*ω* (1)

**Figure 1:**
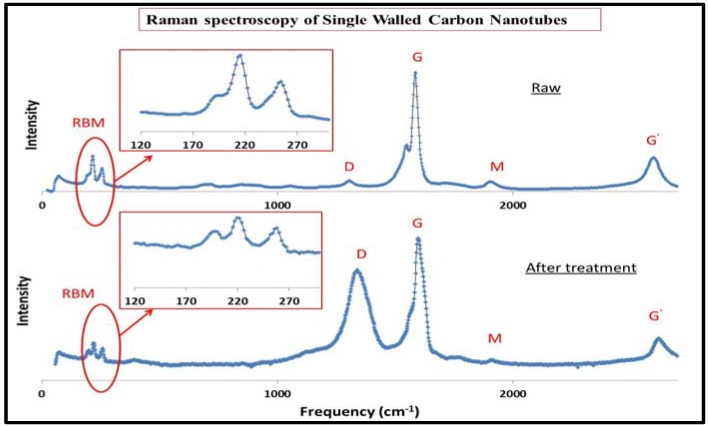
Raman spectra of single-walled carbon nanotubes before and after treatment by 6 M HNO_3_ for 6 h at 120 °C. RBM, radial breathing mode.

Figure 1 shows the Raman spectra of raw single-walled carbon nanotubes (SWCNT) and after acid treatment with 6 M HNO_3_. The intensity of the G band in raw SWCNT is considerably higher than the D band, due to the disorder-induced phonon mode of the carbon six-fold ring breathing vibration. On the other hand, the intensity of the G band after acid treatment decreases, and the D band intensity slightly increase. The higher ratio explains the higher amorphous carbon content and defect formation. The intensity ratios of the D and G bands of the raw and treated CNT samples are 0.0615 and 0.7452, respectively (Table 1). It is worth mentioning that the M band almost completely disappeared. This observation leads to the conclusion that SWCNT after acid treatment has low amorphous carbon content and defects. Figure 1 also shows the RBM band of the raw and acid-treated SWCNT. It can be observed that the absolute intensities of the radial breathing mode are drastically reduced after oxidation. The length of the raw SWCNT decreased after acid treatment; from 1.30–1.27 nm, 1.16–1.13 nm and 0.98–0.96 nm, respectively, as shown in Table 1. These changes indicate chemical alteration of the SWCNT.

### 3.2. Characterization of SWCNT after Functionalization by Visible Spectrophotometry

**Figure 2:**
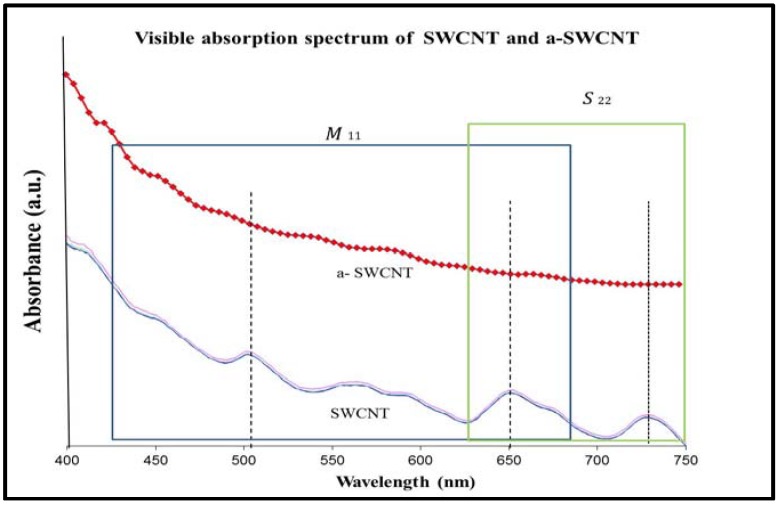
The visible absorption spectra of dispersions containing 0.2% *w*/*v* pristine SWCNT and a-SWCNT, which were measured after a 20-fold dilution.

Visible spectrophotometry was also used to investigate the SWCNT surface after acid treatment. Visible spectrophotometry in the wavelength range from 400–750 nm was used to monitor sidewall perturbation of SWCNT before and after nitric acid treatment [37,38]. The visible absorption spectra of SWCNT and acid-treated SWCNT (a-SWCNT) samples are shown in Figure 2. The boxes in the figure represent the approximate boundaries for metallic and semiconducting transitions (the van Hove transitions). For metallic nanotubes, *M*_11_ represents the electronic transitions between the valence band and conduction band, and in semiconducting nanotubes, *S*_22_ is the electronic transition between the second valence to conduction bands [39,40]. The SWCNT sample shows three distinct peaks at 505, 660 and 730 nm, and they correspond to the second pair of van Hove singularities of the semiconducting and metallic nanotubes [38,41,42,43]. The a-SWCNT sample does not show any distinct peaks in Figure 2. This implies that nitric acid treatment introduces structural defects or functional groups on SWCNT, which result in electronic band transition changes [38]. The changes observed in the van Hove singularities vanish in the spectra, and can be attributed to re-hybridization at carbon (sp^2^ to sp^3^) because the π electrons in the highest occupied molecular orbitals (HOMOs) are used to form new bonds to accommodate the carboxyl group [37,38,43].

### 3.3. Dispersion of a-SWCNT in DMF

**Figure 3:**
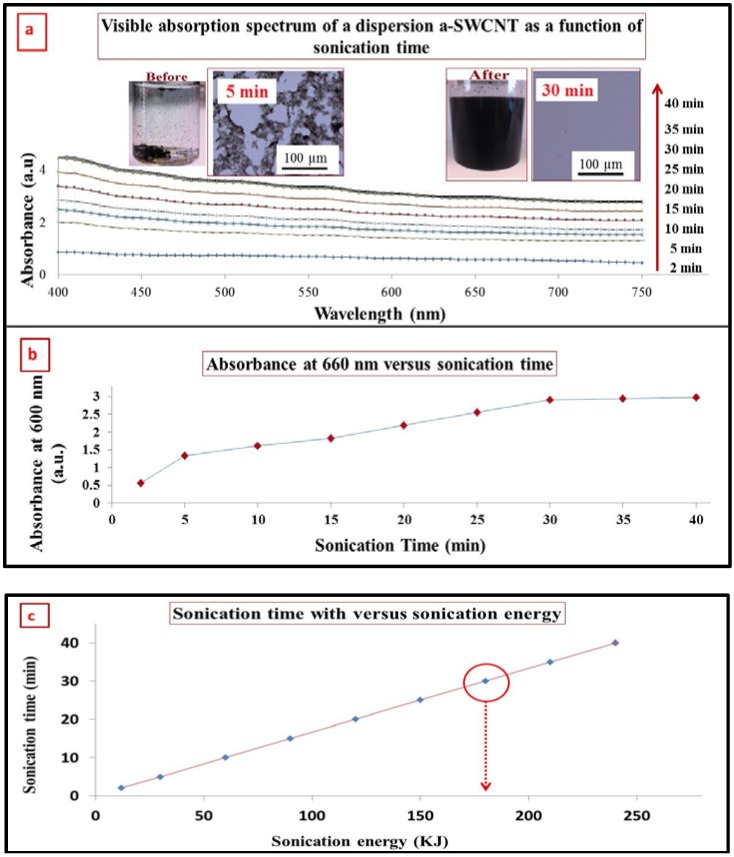
Effect of increasing sonication time on: (**a**) the visible absorption spectrum of a dispersion containing 0.2% *w*/*v* a-SWCNT, which was measured after a 20-fold dilution, where the inset photo images are of 0.2% *w*/*v* containing a-SWCNT before sonication and after 30 min of sonication and the optical microscope image after 5 min and 30 min sonication time; and (**b**) absorbance at 660 nm. (**c**) Effect of increasing sonication time on sonication energy. Arrows in (**a**) indicate the direction of increase in sonication time.

The optimum time of sonication required to effectively disperse CNTs needs to be determined, since excess sonication can shorten or create defects in the tubes that are detrimental to their inherent properties [44,45]. Visible spectroscopy can be used to study the dispersal process by monitoring the variation in absorbance during dispersal to determine the effectiveness of the dispersion and subsequently the stability [41,42,46]. In order to investigate the effect of sonication time on the dispersion of a-SWCNT in DMF, the sample was prepared as a 15 mL dispersion containing a-SWCNT 0.2% *w*/*v* in DMF; which is one of the best solvents reported for dispersing SWCNT [29]. In this experiment, a high concentration was used with a view toward achieving high loading of a-SWCNT on the RVC electrodes. This leads to an increase in the viscosity of the solution, which impedes efficient dispersion. Therefore, an increase in energy is needed to counteract this. Figure 3a,b shows the visible spectra of a-SWCNT dispersions as a function of sonication time, and the corresponding optical absorbance at λ (660 nm) was plotted as a function of sonication time, respectively. It can be seen that the a-SWCNT spectra become more pronounced (higher absorbance) with longer sonication time, indicating that an increasing amount of a-SWCNT became dispersed with time. However, the absorption of the dispersion did not change dramatically after sonication beyond 30 min, indicating that the solution was saturated. The minimum amount of time required to effectively disperse the a-SWCNT was 30 min. The inset images in Figure 3a suggest that homogenous dispersions have been obtained after 30 min sonication time. Furthermore, it was observed that when the sonication time was increased, the color of the solution became progressively black. From Figure 3c, the minimum amount of energy required to effectively disperse 30 mg of the a-SWCNT was 180 KJ at 30 min. This means that 6 kJ was expended per mg of a-SWCNT in a 0.2% *w*/*v* solution. The sonication energy in Figure 3c was calculated using Equation (2).

*E* = *Pt* (2)

where *E* is the sonication energy in joules, *P* is the sonication power (Watts) and *t* is sonication time(s). In this experiment, the sonication power output is 100 W.

### 3.4. Optimization of RVC Electrodes Coated with a-SWCNT

**Figure 4:**
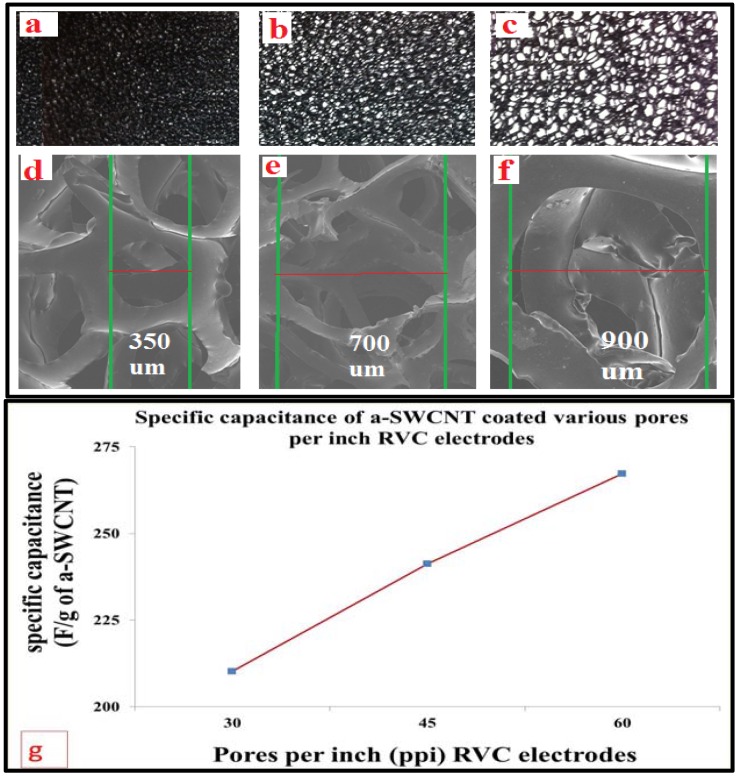
(**a**–**c**) Photo images and (**d**–**f**) SEM micrographs of 60, 45 and 30 ppi RVC samples, respectively; (**g**) the specific capacitance of a-SWCNT-coated RVC electrodes of various porosities in 1 M NaCl solution calculated from cyclic voltammograms recorded in a voltage range between −0.2 and 1.0 V using a three-electrode system vs. Ag/AgCl at a 5-mV/s scan rate.

The general purpose of this work is the optimization of the reticulated vitreous carbon (RVC) electrodes of different porosities coated with a-SWCNT. RVC electrode has a free void volume between 90% and 97%. Thus, RVC electrodes have a low flow resistance. Figure 4a–c shows photo images of three RVC electrodes with porosities of 60, 45 and 30 ppi. It is clear that the free void volume of the RVC electrodes increases with decreasing ppi grade. The average pore sizes of the RVC electrodes were calculated from SEM images by measuring the distance between green lines in Figure 4d–f, and they were 350, 700 and 900 µm for 60, 45 and 30 ppi, respectively. RVC electrode properties are dependent on the ppi grade. If the amount of pores per inch (ppi) increases, the electrode area per unit electrode volume will increase, as well. According to previous reported properties of RVC [47,48], which are good surface area, conductivity and good mechanical strength, it is envisaged that the RVC electrode that has the largest number of pores per inch would be the best to be used as an efficient electrode. Therefore, in order to confirm that the smaller pores electrode is the best electrode for loading of a-SWCNT, we investigated the influence of all electrode capacitances of RVC electrodes with different pore sizes coated with the same amount of a-SWCNT. All RVC electrodes had the same geometric volume (dimensions of 4.0 cm × 1.8 cm × 0.3 cm), and the same amount of a-SWCNT was coated, around 6 mg. The effect of different pores per inch was investigated in aqueous solution. Figure 4g shows the capacitances of all electrodes that were calculated from cyclic voltammograms at the potential scan rate of 5 mV/s using the following Equation (3) [49];

*C*_mass_ = *Q*/(2*m*Δ*V*) (3)

where *C*_mass_ is the capacitance of electrode (F/g), *Q* is charge (C), *m* is mass (g) and *V* is voltage (V). It can be seen that the highest specific capacitance was 267.24 F/g for the a-SWCNT-coated RVC electrode with 60 pores per inch, and the specific capacitance decreased with the decrease in the amount of pores per inch. This is because the RVC electrode with 60 ppi has a higher surface area per volume of electrode. The RVC 60 ppi electrode coated with a-SWCNT (6 mg) will be discussed in detail in its electrochemical behavior evaluation using cyclic voltammetry. In conclusion, therefore, 60 ppi RVC electrodes were selected as substrates to load a-SWCNT for use as electrode material.

### 3.5. Optimization of the Loading Level of a-SWCNT on the RVC Electrode

**Figure 5:**
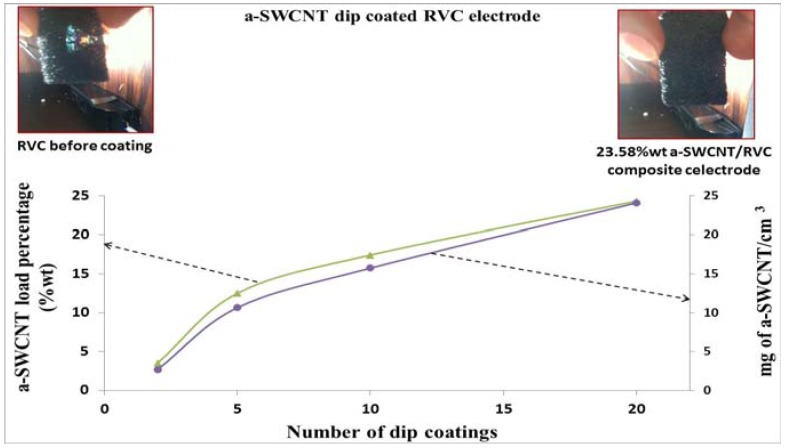
a-SWCNT loading of RVC as a function of the number of dip coatings in the a-SWCNT dispersion. Insets: Images of the RVC electrode before coating and after being completely filled (23.58 wt % a-SWCNT-coated RVC electrode).

Figure 5 shows that the percentage of loading of a-SWCNT increased with repeated electrode immersion in the a-SWCNT solution. RVC electrodes, Numbers 1, 2, 3 and 4, were repeatedly immersed in the solution 2, 5, 10 and 20 times, respectively, and the amounts of a-SWCNT loaded onto RVC to form a-SWCNT/RVC composite electrodes were found to be 6 mg (3.63 wt %), 23 mg (12.50 wt %), 34 mg (17.43 wt %) and 50 mg (23.85 wt %), respectively (Table 2). In the laboratory, it was observed that the pores of this RVC electrode at 23.85 wt % loading were completely filled by a-SWCNT as seen in the inset image in Figure 5 when tested with a light. All these electrodes, however, allowed liquid to flow through because the SWCNT filling itself is porous.

### 3.6. Scanning Electron Microscopy of a-SWCNT-Coated RVC Electrodes

**Figure 6:**
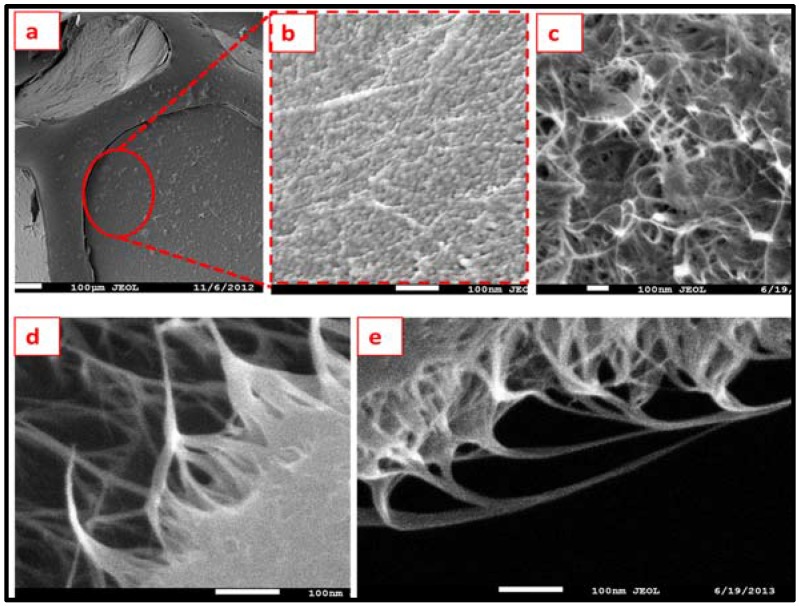
Scanning electron microscopy (SEM) images (**a**,**b**) top surface, (**c**) cross-section and (**d**,**e**) 45° view of 23.58 wt % a-SWCNT loading.

The surface morphology of the a-SWCNT-coated RVC electrode was examined using scanning electron microscopy (SEM). SEM images of the 23.58 wt % a-SWCNT-coated RVC electrode are shown in Figure 6. It can be concluded that the a-SWCNT dispersed very well in DMF because no aggregation is seen on the surface and RVC pores appeared filled completely (Figure 6a). The appearance of the top surface is like textile (Figure 6b) because the nanotubes are irregularly spread on the RVC electrode and its pores, partly parallel and partly perpendicular to the surface. The SEM images in Figure 6c–e shows that the a-SWCNT tubes became stronger and tougher due to the closer contact, which improved ion transfer between nanotubes, which is consistent with previously-reported results [50,51]. In addition, it shows that the void spaces or “pores” between the matted SWCNT tubes are macroscale, and these void spaces can be considered as macropores and are areas through which ions diffusion can freely take place. From the SEM image, SWCNT nano-network structures act as useful nano-spacers for diminishing the aggregation of SWCNT. This 3D porous structure exposes an extensive surface area that facilitates ions’ diffusion, leading to a high performance of electrosorption. It maximizes the surface area, potentially allowing large capacitances to be obtained [52]. This also will lead to an increase in ions’ capture and the conductive properties of a-SWCNT.

### 3.7. Electrochemical Behavior Evaluation Using Cyclic Voltammetry

In order to evaluate the electrochemical properties of the a-SWCNT-coated RVC electrode, cyclic voltammetry was applied as it is one of the most widely-used techniques to study electrochemical reactions. In this section, it is used to determine the effect of filling the pores of RVC by a-SWCNT on the capacitance, the effect of increasing scan rate on electron transfer and capacitance and the stability of the electrode using 1 M NaCl solution recorded in the voltage range between −0.2 and 1.0 V vs. Ag/AgCl in a three-electrode system with an RVC counter electrode.

### 3.8. The Capacitance of RVC Electrode before and after Loading with a-SWCNT 

The capacitance of the RVC electrode in 1 M NaCl solution using three-electrode systems was discussed in our previous publication [53], and it was calculated to be 0.002 F/cm^3^ using the following Equation (4) [49].

**Figure 7:**
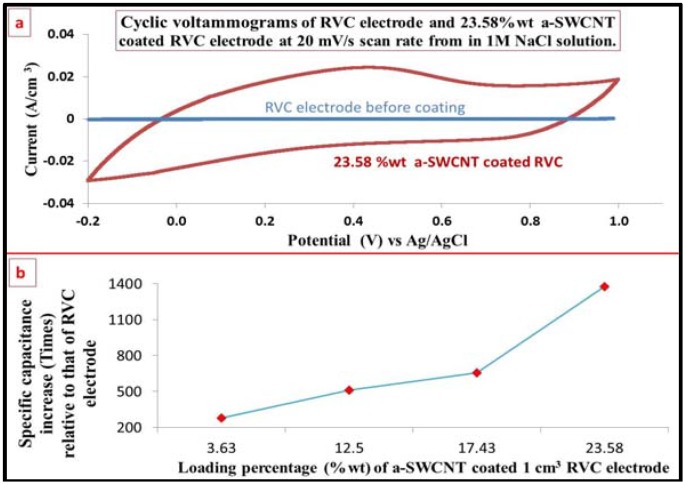
(**a**) Comparison cyclic voltammograms of the 1 cm^3^ bare RVC electrode and the same size of the 23.58 wt % a-SWCNT-coated RVC electrode in 1 M NaCl scanned at 20 mV/s and using an RVC counter electrode and Ag/AgCl reference electrode in a three-electrode system; and (**b**) the effect of a-SWCNT loading of a-SWCNT/RVC composite electrodes on specific capacitance.

*C*_volume_ = *Q*/(2*Z*Δ*V*) (4)

where *C*_volume_ is the capacitance of electrode (F/cm^3^), *Q* is charge (C), *Z* is geometric volume (cm^3^) and *V* is voltage (V). Figure 7a shows the cyclic voltammograms of the 1 cm^3^ RVC electrode and the same electrode filled with 23.58 wt % a-SWCNT under the same conditions. The peak current (i_p_) of the 23.58 wt % a-SWCNT-coated RVC electrode compared to a bare RVC electrode has increased by a factor of 1375. This is related to the large surface area of a-SWCNT compared to a bare RVC electrode according to the Randle–Sevcik relationship [54]. The specific capacitance of the 23.58 wt % a-SWCNT-coated RVC electrode was 2.75 F/cm^3^. Figure 7b shows that the specific capacitance of the electrode increased with increasing amounts of a-SWCNT in geometric volume. The specific capacitance was 0.56, 1.02 and 1.31 F/cm^3^ for the electrode that had 3.63, 12.50 and 17.43 wt % a-SWCNT coated on the RVC electrodes, respectively. The specific capacitances of 3.63, 12.50 and 17.43 wt % a-SWCNT coated on the RVC electrodes compared to a bare RVC electrode have increased by a factor of 280, 510 and 655, respectively. Figure 7a shows the pseudo-capacitive behavior at 0.5 V for the RVC electrode coated by a-SWCNT. This behavior referred to the functional group, which was created by the nitric acid treatment on the SWCNT surface [55].

### 3.9. Effect of Increasing the Scan Rate on the Electrode Capacitance

**Figure 8:**
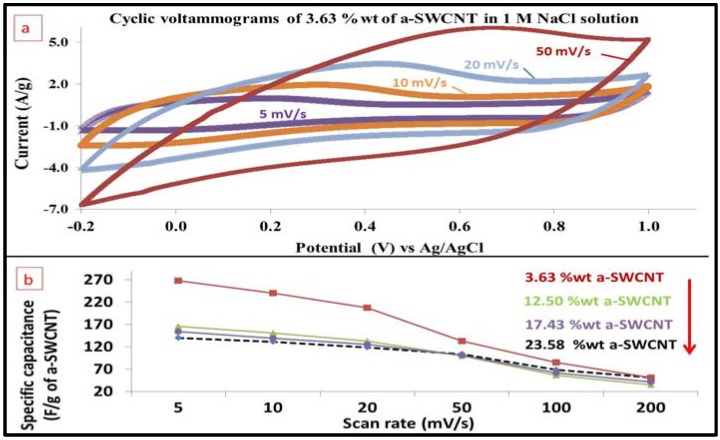

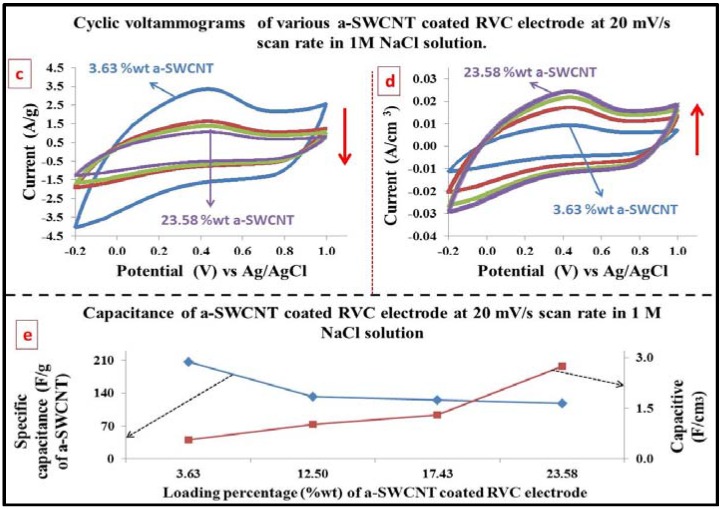
(**a**) Cyclic voltammograms of the 3.63 wt % a-SWCNT-coated RVC electrode and (**b**) specific capacitance of various a-SWCNT-coated RVC electrodes at various scan rates in 1 M NaCl solution in the voltage range between −0.2 and 1.0 V vs. Ag/AgCl using a three-electrode system. Cyclic voltammograms of a-SWCNT-coated RVC electrodes at 20 mV/s, in terms of (**c**) current per gram of a-SWCNT and (**d**) current per geometric volume of electrode; (**e**) Capacitance of the electrodes per gram of a-SWCNT and per geometric volume of the electrode.

The capacitive behavior of the a-SWCNT resulted mainly from electrochemical double-layer charging along with a negligible contribution of pseudo-capacitance. In this study, the effect of different scan rates on electrode capacitance was investigated in 1 M NaCl aqueous solution. Figure 8a shows the cyclic voltammograms of the 3.63 wt % a-SWCNT-coated RVC working electrode obtained with various potential scan rates. It can be noted that electrochemical reactions occur in the potential range of (−0.2–0.4 V) for scan rates of 5 mV/s, resulting in redox peaks at about 0 and 0.2 V. However, the shapes of the CVs at scan rates of 5–20 mV/s are close to rectangular, and as we know, the achievement of a rectangular-shaped CV is the suggested ultimate goal in electrochemical double-layer capacitors (EDLC) [49]. The absence of Faradaic reactions at 0.5 V indicates that the current response here absolutely comes from the electric double layer EDL formation [56]. The enhanced capacitance compared with raw SWCNT relates to the increased surface charge density from oxygen atoms with more negative electronic affinity [55]. According to the characteristics of the CV, it can be deduced that the contribution of carboxyl and carbonyl groups to CNTs is in the pseudo-capacitance, which increases apparent capacitance [15,57]. It is clear that, the polarization caused by a high scan rate leads to the anodic peaks shifting toward high potential and the cathodic peaks moving toward negative potential simultaneously [15]. However, when the scan rate was increased to 50 mV/s, the curves were characterized by a non-rectangular shape that indicated resistance-like electrochemical behavior. This leads to a continuous decrease in the capacitance of electrodes with increasing scan rate.

Figure 8b presents the capacitances of various a-SWCNT-coated RVC electrodes as a function of the scan rate. It is observed that increasing the amount of a-SWNT in the RVC electrode led to a decrease in the specific capacitance of a-SWCNT. For example, the highest specific capacitance was 267.24 F/g for 3.63 wt % a-SWCNT, and the lowest specific capacitance was 139.65 F/g for 23.58 wt % a-SWCNT at a scan rate of 5 mV/s. It seems also that a-SWCNT-coated RVC electrodes gave, in all electrodes, a high capacitance at a low scan rate, but its capacitance markedly decreased at high scan rates. For instance, the specific capacitance of 3.63 wt % a-SWCNT coated on RVC was 267.24, 239.58, 207.40, 132.69, 84.77 and 51.15 F/g at 5, 10, 20, 50, 100 and 200 mV/s, respectively (Table 3). It is expected that the capacitive volume should increase with increasing scan rates because it is found in all cases of carbon nanotubes [15,49,55,56,58]. This characteristic has been attributed to the resistance of the electrolyte and the inner resistance of ion diffusion with certain carbon micro-pores being surface partially accessible to electrolytes. This becomes significant under relatively high scan rates due to the differential depletion of the electrolyte concentration [58,59,60]. It can be seen that the specific capacitance trend of the 3.63 wt % electrode sharply decreases when the scan rate is above 20 mV/s. For other electrodes, the specific capacitance trend decrease is more pronounced, when the scan rate is increased above 50 mV/s. The specific capacitance values of all electrodes were calculated using Equation (3).

Figure 8c,d shows cyclic voltammograms of a-SWCNT composite electrodes with different loadings of a-SWCNT in terms of current per gram of a-SWCNT and current per geometric volume of electrode, respectively. It is observed that increasing the amount of a-SWNT in the RVC electrode led to a decrease in the current per gram of a-SWCNT (Figure 8c) but, in contrast, led to an increase in the current per geometric volume of the electrode (Figure 8d). Hence, the effect of the geometric volume of the electrode on the results will now be discussed. The calculated capacitance per unit geometric volume of porous electrode is presented in Figure 8e and Table 3 as F/cm^3^. This is not the same as per unit volume of active material (i.e., SWCNT) alone. The capacitance of the electrode per geometric volume (F/cm^3^) was calculated using Equation (4). Figure 8e shows that the capacitances of various a-SWCNT-coated RVC electrodes increase with the increase in the amount of a-SWCNT in the geometric volume. The specific capacitance, obtained at 20 mV/s, was 0.56, 1.02, 1.31 F/cm^3^ and 2.75 F/cm^3^ for the electrode that had 3.63, 12.50, 17.43 and 23.58 wt % a-SWCNT coated on the RVC electrodes, respectively. This indicates that the surface area of a-SWCNT coated on the RVC electrode had increased. In addition, the effect of the geometric area of the electrode on the results will now be considered. The calculated capacitance per unit geometric area of porous electrode is presented in Table 3 as F/cm^2^. The capacitance of electrode per geometric area (F/cm^2^) was calculated using Equation (5), which can be given as [49];

*C*_area_ = *Q*/(2*A*Δ*V*) (5)

where *C*_area_ is the capacitance of the electrode (F/cm^2^), *Q* is charge (C), *A* is geometric area (cm^2^) and *V* is voltage (V). The increase of SWCNT loading level could increase the active area resulting in higher specific capacitance. In Table 3, the capacitance obtained at 20 mV/s was 0.07, 0.12, 0.16 and 0.33 F/cm^2^ for the electrode that had 3.63, 12.50, 17.43 and 23.58 wt % a-SWCNT coated on the RVC electrodes, respectively. This indicates that the surface area of a-SWCNT coated on the RVC electrode had increased.

### 3.10. Electrochemical Impedance Spectroscopy 

**Figure 9:**
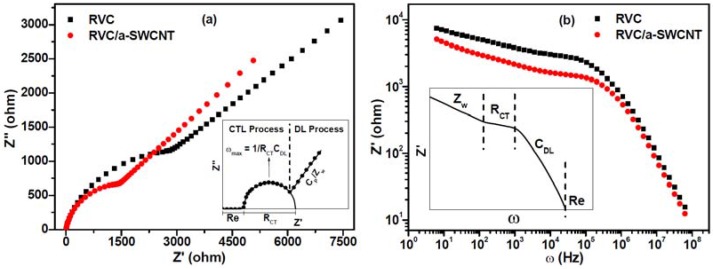
(**a**) Nyquist plot (inset diagram) and (**b**) Bode plot of RVC and the RVC/a-SWCNT composite electrode.

The electrochemical impedance spectra of bare RVC and RVC/a-SWCNT (23.58 wt %) composite electrode are presented in Figure 9. Figure 9a represents the Nyquist plots of both electrodes where the real part of impedance (*Z*′) is plotted against the imaginary part of impedance (*Z*″). Both plots show semicircles at the higher frequency region, and straight spikes at the lower frequency region imply a charge-transfer limited (CTL) process (also known as redox process) and a diffusion limited (DL) process (also shown diagrammatically in the inset of Figure 9a), respectively. The obtained semicircular shape at the higher frequency region is due to the polarization of electrodes; whereas the diameter of the semicircle accounts for the polarization/charge-transfer resistance. This polarization resistance concerns the porous structure of the electrode and the ion diffusion through its macro pores. It is observed that the diameter of the semicircle for the RVC/a-SWCNT composite electrode is less compared to the bare RVC electrode, indicating the low resistive behavior of the RVC/a-SWCNT composite electrode compared to the bare RVC one. The reduction in semicircle diameter for the RVC/a-SWCNT composite electrode also indicates the high ionic infiltration/charge-transfer on its surface. The intersection of the semicircle with the x-axis at high and middle frequency regions gives the value of electrolyte resistance (*R*_e_) and charge-transfer resistance (*R*_CT_), respectively, as shown diagrammatically for better understanding in the inset of Figure 9a. Accordingly, the Re and R_CT_ values for bare RVC electrode are 15.4 ohm and 5203 ohm, respectively; whereas for the RVC/a-SWCNT electrode, the values are 12.3 ohm and 2675 ohm, less compared to the bare RVC electrode. This can be attributed to the fact that the hindrance to the charge transfer process is minimized for the RVC/a-SWCNT composite electrode because of the increase in the number of conductive paths due to CNTs entanglements after coating a-SWCNT on RVC. The value of double layer capacitance (C_DL_) can be calculated from the relation *ω*_max_ = 1/(*R*_CT_*C*_DL_), where *ω* is the angular frequency. The calculated values of *C*_DL_ for the RVC electrode and the RVC/a-SWCNT electrode are 3.6 and 4.9 nF. Thus, an increment in the C_DL_ value is observed after coating RVC with a-SWCNT because of the increase in surface area that facilitates diffusion through the macrospores, leading to the high performance of electrosorption.

The real impedance at low frequencies, where the impedance diagrams show straight spike inclination around 45°, is attributed to the dominance of diffusion-limited Warburg impedance (*Z*_w_)/pseudo capacitance (*C*_P_) due to limited mass transport of redox species from the bulk of solution to the electrode and ionic/charge accumulation on the surface of the electrode. It is seen from the Figure 9a that the inclination of straight spike is relatively more for the RVC/a-SWCNT electrode compared to the RVC electrode, indicating that the former has a high value of pseudo capacitance. This can be attributed to the presence of the carboxyl and carbonyl group on the surface of CNTs.

The impedance results can be represented by another means using the Bode plot, where the real part of impedance (*Z*′) is plotted against angular frequency (*ω*) as shown in Figure 9b for both RVC and RVC/a-SWCNT electrodes. The Figure 9b shows a high value of impedance for the RVC electrode compared to the RVC/a-SWCNT electrode over the entire frequency region. The low impedance value for the RVC/a-SWCNT electrode is due to the interconnected CNT-CNT network on the surface of the electrode, which facilitates both ionic and electronic transport through its network. It is interesting to show which regions of the curves represent the different parameters. Accordingly, a diagram has been drawn and is shown in the inset of Figure 9b for better understanding. Warburg impedance (*Z*_w_) is seen in the low frequency region, identified as inclined nearly −45°; charge transfer resistance (*R*_CT_) in the middle frequency region is identified on the basis of the horizontal line close to 0°; double layer capacitance (*C*_DL_) in the high frequency region is identified around an inclination of −90°; and finally, electrolyte resistance (*R*_e_) at a high frequency gives the horizontal line at 0°. Hence, it can be said that the results and diagram of the Nyquist and Bode plots resemble each other.

### 3.11. Cycling Stability of a-SWCNT/RVC Electrodes

**Figure 10:**
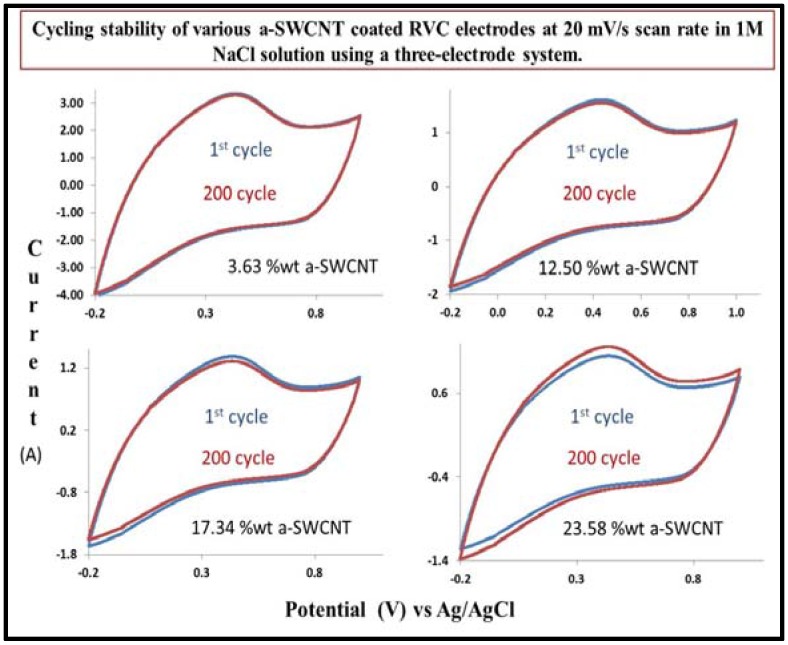
The electrochemical cycling stability of a-SWCNT-coated RVC electrodes, with different a-SWCNT loadings, in 1 M NaCl solution recorded after 200 cycles in the voltage range between −0.2 and 1.0 V vs. Ag/AgCl using a three-electrode system at a 20-mV/s scan rate.

The electrochemical cycling performance of the electrodes with various amounts of a-SWCNT coated on the RVC electrode was investigated for 200 cycles by cyclic voltammetry, as shown in Figure 10. It is clear that they all exhibit excellent cycling performance during the first 200 cycles, and the results show that a lower amount of a-SWCNT on the RVC electrode exhibits a much better stability. After 200 cycles, the 3.63, 12.50, 17.43 and 23.58 wt % a-SWCN-coated RVC electrodes maintained 98, 95, 94 and 91% of their capacitances, respectively. The main initial loss of stability can be attributed to the decrease in the redox activity of the redox process. The decrease in the capacitance of each electrode can be attributed to the decrease of the pore structure of the electrode and to the increase of the ratio of C to O attributed to the phenolic hydroxyl groups on the electrodes [57]. Consequently, the capacitance decreased with the cycle number due to the increase in the number of oxygen-containing functional groups on the electrode. The results show that a lesser amount of a-SWCNT on the RVC electrode exhibits a much higher discharge capacity and excellent cycling stability.

## 4. Conclusions

The analysis through Raman spectra reveals that acid-treated SWCNTs have low amorphous carbon content and defects compared to untreated ones. Moreover, it has been proven through visible spectra that nitric acid treatment introduces structural defects or functional groups on SWCNT, which result in electronic band transition changes. The optimum sonication time for proper dispersion of SWCNTs in DMF was found to be 30 min, which means sonication energy of 6 kJ was expended per mg of a-SWCNT. The average pore size was found to be 350, 700 and 900 µm for RVC electrodes with porosities of 60, 45 and 30 ppi, respectively. The investigation confirmed that the RVC electrode with a porosity of 60 ppi was the best electrode for loading of a-SWCNT because the a-SWCNT-coated RVC electrode showed the highest specific capacitance value compared to other tested electrodes. This is because the RVC electrode with a porosity of 60 ppi had a higher surface area per volume of electrode. The amount of a-SWCNT on RVC increases with the increase in the number of immersions into the solution, and it was observed that when the pores of RVC were completely filled with a-SWCNT, the amount of a-SWCNT in the composite was 23.85 wt %. The SEM study shows that the pores in between the matted a-SWCNT are in macroscale, which means macropores through which the ions can diffuse freely. The increment in specific capacitance for 3.63, 12.50 and 17.43 wt % a-SWCNT coated on the RVC electrodes compared to a bare RVC electrode was by a factor of 280, 510 and 655, respectively. It was shown that there is a decreasing trend of specific capacitance for 3.63 wt % a-SWCNT coated on RVC electrode, which was 267.24, 239.58, 207.40, 132.69, 84.77 and 51.15 F/g at the increasing scan rates of 5, 10, 20, 50, 100 and 200 mV/s, respectively. However, the value of specific capacitance per geometric volume (obtained at 20 mV/s) for 3.63, 12.50, 17.43 and 23.58 wt % a-SWCNT coated on the RVC electrodes was 0.56, 1.02, 1.31 and 2.75 F/cm^3^, respectively. This indicated that the surface area of a-SWCNT coated on the RVC electrode had increased. All the electrodes showed good cyclic stability; however, the stability is better for lower a-SWCNT-loaded composite electrodes. The above results indicate that these composites can be effective materials for desalination technology.

## Figures and Tables

**Table 1:** Single-walled carbon nanotube (SWCNT) diameter and ratio of D to G bands before and after acid treatment.

(SWCNT)	ω (cm^−1^)	*d_t_* (nm)	I_D_/I_G_
Raw	191	1.30	0.0615
	213	1.16	
	254	0.98	
Treated	196	1.27	0.7452
	219	1.13	
	258	0.96	

**Table 2:** RVC (2.16 cm^3^) electrodes before and after a-SWCNT coating.

Sample	1	2	3	4
RVC (mg)	159	161	161	162
After coating (mg)	165	184	195	212
Amount of a-SWCNT (mg) in sample	6	23	34	50
Amount of a-SWCNT in sample (% *w*/*w*)	3.63	12.50	17.43	23.58

**Table 3:** Specific capacitance of various a-SWCNT-coated RVC electrodes by F/g of a-SWCNT, F/area of electrode and F/volume of electrode, in 1 M NaCl solution at various scan rates.

**Sample**		**3.63 wt % a-SWCNT-Coated RVC**					
Scan rate (mV/s)		5	10	20	50	100	200
Specific Capacitance	(F/g)	267.24	239.58	207.40	132.69	84.78	51.15
	(F/cm^2^)	0.09	0.08	0.07	0.03	0.03	0.02
	(F/cm^3^)	0.72	0.64	0.56	0.36	0.23	0.14
**Sample**		**12.50 wt % a-SWCNT-Coated RVC**					
Scan rate (mV/s)		5	10	20	50	100	200
Specific Capacitance	(F/g)	165.20	150.33	132.16	99.12	56.17	34.69
	(F/cm^2^)	0.21	0.18	0.12	0.07	0.04	0.02
	(F/cm^3^)	1.76	1.46	1.02	0.55	0.31	0.17
**Sample**		**17.43 wt % a-SWCNT-Coated RVC**					
Scan rate (mV/s)		5	10	20	50	100	200
Specific Capacitance	(F/g)	153.79	138.94	125.20	100.77	61.07	41.23
	(F/cm^2^)	0.29	0.23	0.16	0.08	0.04	0.02
	(F/cm^3^)	2.42	1.93	1.31	0.67	0.37	0.19
**Sample**		**23.58 wt % a-SWCNT-Coated RVC**					
Scan rate (mV/s)		5	10	20	50	100	200
Specific Capacitance	(F/g)	139.65	131.27	117.70	103.34	68.43	51.67
	(F/cm^2^)	0.39	0.37	0.33	0.29	0.19	0.14
	(F/cm^3^)	3.23	3.04	2.75	2.39	1.58	1.20
